# TAVI in a Heart Transplant Recipient—Rare Case Report and Review of the Literature

**DOI:** 10.3390/biomedicines11102634

**Published:** 2023-09-26

**Authors:** Silvia Preda, Lucian Câlmâc, Claudia Nica, Mihai Cacoveanu, Robert Țigănașu, Aida Badea, Alexandru Zăman, Raluca Ciomag (Ianula), Claudiu Nistor, Bogdan Severus Gașpar, Luminița Iliuță, Lucian Dorobanțu, Vlad Anton Iliescu, Horațiu Moldovan

**Affiliations:** 1 Faculty of Medicine, Carol Davila University of Medicine and Pharmacy, 050474 Bucharest, Romania; dr.silvia.preda@gmail.com (S.P.); raluca.ianula@umfcd.ro (R.C.); ncd58@yahoo.com (C.N.); bogdan.gaspar@umfcd.ro (B.S.G.); luminitailiuta@yahoo.com (L.I.); vladanton.iliescu@gmail.com (V.A.I.); 2 Department of Cardiovascular Surgery, Bucharest Clinical Emergency Hospital, 014461 Bucharest, Romania; lcalmac@gmail.com (L.C.); bianca.nica@yahoo.com (C.N.); tiganasu.robert@yahoo.com (R.Ț.); aidafirtade@gmail.com (A.B.); alexandrusebastianzaman@gmail.com (A.Z.); 3 Department of Cardiology, “Bagdasar Arseni” Clinical Emergency Hospital, 041915 Bucharest, Romania; 4 Department of Thoracic Surgery, Central Military Emergency University Hospital, 013058 Bucharest, Romania; 5 Cardioclass Clinic for Cardiovascular Disease, 031125 Bucharest, Romania; 6 Faculty of Medicine, Titu Maiorescu University, 040441 Bucharest, Romania; lucian.dorobantu@prof.utm.ro; 7 Department of Cardiovascular Surgery, Monza Metropolitan Hospital, 040204 Bucharest, Romania; 8 Department of Cardiovascular Surgery, Prof. Dr. C.C. Iliescu Emergency Institute for Cardiovascular Diseases, 022322 Bucharest, Romania; 9 Academy of Romanian Scientists, 54, Spl. Independentei, 050711 Bucharest, Romania

**Keywords:** orthotopic heart transplantation (OHT), transcatheter aortic valve implantation (TAVI), heart valve replacement, aortic valve

## Abstract

The global demand for cardiac transplants continues to rise, even with advancements in assistive devices. Currently, the estimated annual mortality rate stands at 3–5%, and patients often face a waiting time of approximately four years on transplant waiting lists. Consequently, many transplant centers have started to consider heart transplants from donors who may be deemed “less than ideal” or marginal. However, the decision to accept such donors must be highly individualized, taking into consideration the risks associated with remaining on the waiting list versus those posed by the transplantation procedure itself. A potential solution lies in the creation of two distinct recipient lists, matched with donor criteria, allowing marginal donors to provide the lifeline that selected patients require. This paper follows a two-step approach. Firstly, it offers an overview of the current state of affairs regarding the topic of transcatheter aortic valve implantation (TAVI) in orthotopic heart transplant (OHT) patients. Secondly, it presents firsthand experience from our clinical center with a comprehensive case presentation of a patient in this unique medical context. The clinical case refers to a 62-year-old male patient, a smoker with a history of hypertension, dyslipidemia, and a prior OHT a decade earlier, who presented with fatigue during minimal physical exertion. The Heart Team carefully reviewed the case, considering the patient’s immunosuppressed status and the heightened risk associated with a repeat intervention. In this instance, transcatheter aortic valve implantation (TAVI) was deemed the appropriate treatment. The TAVI procedure yielded successful results, leading to improved clinical status and enhanced cardiac function. The inclusion of marginal donors has introduced novel challenges related to the utilization of previously diseased marginal organs. TAVI has already demonstrated its efficacy and versatility in treating high-risk patients, including heart transplant recipients. Consequently, it emerges as a vital tool in addressing the unique challenges posed by the inclusion of marginal donors.

## 1. Background

In the present day, orthotopic heart transplantation (OHT) stands as one of the established approaches for managing advanced heart failure. Its legacy spans over 55 years, commencing with the controversial inaugural global human transplant in 1967 [1].

Much later, in 2002, the inaugural transcatheter aortic valve implantation (TAVI) procedure was successfully conducted by Professor Alain Cribier in France. The development of this technique began in 1980 due to an increasing demand for alternative treatments. In its early stages, the primary focus was on balloon aortic valvuloplasty for patients with high-risk aortic stenosis. Although this approach led to symptom improvement, it was associated with numerous complications and lacked favorable mid- to long-term outcomes. As a result, it is now considered only as a palliative measure when TAVI or surgical aortic valve replacement (SAVR) is not feasible.

In 1992, the first crimped biological valve prosthesis was patented, though it remained unused until further studies in 1994 demonstrated its ability to maintain its shape within a calcified annulus. Despite initial skepticism surrounding TAVI, multiple studies conducted between 1999 and 2002 affirmed its viability as a valuable treatment option [2].

Since its inception, OHT has witnessed significant advancements in patient survival, medical interventions, and the management of associated complications. Notably, the 5-year survival rate following OHT has increased from 62.7% in 1980 to 72.5% in 2014. Data from the registry of the International Society of Heart and Lung Transplantation (ISHLT) indicates that around 21% of patients remain alive at the 20-year mark, with select centers reporting an impressive 20-year survival rate of 55% of patients [3,4].

Complications that arise following OHT play a pivotal role in determining patient outcomes and their susceptibility to subsequent cardiac surgeries. Among the most commonly reported complications are chronic allograft vasculopathy (CAV), malignancies, infections, acute rejection, and renal insufficiency.

CAV is prevalent in approximately one third of patients within their initial five years post OHT, and this incidence surges to over 50% after a decade. Its significance is underscored by the fact that CAV contributes to approximately 10% of annual deaths among recipients. Ten years post OHT, malignancy diagnoses affect 35% of patients, with skin cancer being the most frequently reported type. After the first five years post OHT, malignancy-related mortality stands at approximately 22% annually.

Infections represent a critical complication, with a substantial mortality rate of up to 30% within the first year following OHT. However, this complication tends to decline in subsequent years, potentially attributable to a reduction in immunosuppressive therapy.

Acute rejection assumes particular importance, especially during the initial years post OHT, accounting for approximately 10% of deaths within the first three years.

The incidence of renal insufficiency escalates over time, reaching 30% at the ten-year mark following OHT [4,5].

Since the inception of OHT and TAVI, significant progress has been achieved in the field of cardiovascular surgery. These advancements have expanded the scope of indications, allowing for the application of these procedures even in cases that fall outside the established guidelines under specific circumstances.

Through consistently delivering positive outcomes and actively sharing experiences, the entire medical community can enhance their clinical practice and remain well-informed about the latest developments in this field. This collaborative approach ensures that patients benefit from the most current and effective treatments available [3].

Despite the remarkable advancements in cardiac surgery, it remains imperative to carefully consider patients with OHT and their unique set of risks and comorbidities. This thoughtful approach is essential for enhancing both their survival rates and overall quality of life. Through tailoring medical care and interventions to the specific needs and challenges of OHT patients, healthcare professionals can continue to improve patient outcomes and ensure a better quality of life for this distinct patient population.

## 2. Literature Review

This section delves into a thorough review of the existing literature, encompassing documented cases and studies related to OHT recipients facing aortic stenosis. It elucidates the challenges associated with this unique patient group and highlights instances where TAVI emerged as a viable intervention.

Currently, there is a noticeable absence of specific clinical guidelines for managing aortic stenosis in patients who have previously undergone OHT. The lack of such guidelines leaves clinicians with limited guidance on how to approach aortic stenosis in this unique patient population.

With the success of heart transplantations and the increasing number of OHT recipients, there is a growing need to address the specific medical conditions and complications that arise in this population. Aortic stenosis is not uncommon in this group, and as such, it necessitates specialized consideration.

Patients with a history of OHT present a clinical complexity that requires tailored approaches. Their immunosuppressive status, previous cardiac surgeries, and comorbidities need to be factored into treatment decisions. Aortic stenosis management in this context demands careful evaluation and a nuanced understanding of the associated risks and benefits of various interventions.

While the absence of established guidelines can be challenging, it also presents an opportunity for innovation in patient care. Exploring alternative procedures such as TAVI and evaluating their outcomes in OHT recipients can pave the way for the development of evidence-based protocols that improve patient outcomes and quality of life.

## 3. Objective

In this literature review, our primary aim was to identify previously published studies of individuals with a history of OHT who subsequently developed aortic valve disease necessitating aortic valve replacement. Our analysis focused on investigating the management strategies employed in such cases, specifically examining the choice of valve type (balloon-expandable or self-expandable) and the approach used (femoral artery or transapical). Additionally, we aimed to assess the clinical outcomes associated with these interventions.

## 4. Methods

### 4.1. Eligibility Criteria

For this literature review, we sought studies involving individuals who had previously undergone OHT and subsequently developed aortic valve disease necessitating aortic valve replacement. We conducted a comprehensive search for articles published in English. In the selected articles, we examined demographic information, including population characteristics, sex, age at OHT, age at TAVI, the time elapsed between OHT and TAVI, the type of aortic valve prosthesis used, the procedural approach, and clinical outcomes.

### 4.2. Information Sources

Our search encompassed electronic databases, including Pubmed, Cochrane Library, and EMBASE. Additionally, we explored the PROSPERO registry for any relevant reviews, although none were identified.

### 4.3. Search Strategy

To identify pertinent studies, we conducted electronic searches using keywords such as heart transplant, OHT, heart transplant recipient aortic disease, aortic stenosis, TAVI, TAVR, and aortic valve replacement after heart transplant.

### 4.4. Study Records–Data Management

Upon selecting articles that described aortic valve replacement in OHT recipients, we sought data pertaining to patient characteristics, age at transplantation and at aortic valve replacement, the duration between OHT and aortic valve replacement, prosthesis type and size, procedural approach, clinical status of patients at the time of OHT and/or aortic valve replacement, and outcomes.

- Selection process: Two independent reviewers selected studies adhering to the aforementioned eligibility criteria from the same electronic databases.
- Data collection process: Given the predominance of case reports in the literature, we independently extracted data from these reports.

We retrieved data related to population characteristics, including age at transplantation and aortic valve replacement, gender, and comorbidities. Additionally, we gathered information on the clinical status of patients at the time of OHT and aortic valve replacement, the type and size of the valve utilized, the procedural approach, and defined outcomes encompassing mortality, complications, the necessity for additional interventions, improvements in cardiac function, and post-procedural clinical status.

### 4.5. Outcomes and Prioritization

Our primary outcomes of interest were patient survival and procedure-related complications, including the need for reintervention, bleeding, tamponade, paravalvular leak, coronary complications associated with the procedure, the requirement for a pacemaker, and broader outcomes such as post-procedural contractility, clinical status enhancement, and hospital discharge.

### 4.6. Data Synthesis

Given the relatively small number of patients and their variability, a systematic representation of the targeted patient profile cannot be made.

Due to the incomplete data reported in the articles found and small population with this particular characteristic, we cannot have a statistical analysis of the cases found in the literature. So, we described each case published until the first draft of the manuscript was submitted and compared the data in a table to make it easier to observe.

## 5. Results

The current clinical guidelines do not provide specific recommendations for the management of individuals with aortic stenosis who have previously undergone OHT. Consequently, we recognized the significance of contributing a case study involving a patient who underwent a TAVI procedure following OHT.

While the medical literature contains an extensive body of research discussing OHT and TAVI as separate entities, there is a paucity of literature addressing the intersection of TAVI in OHT recipients. This knowledge gap has resulted in the absence of a consensus regarding the optimal management of this specific patient population. As a result, clinical decisions are typically made on a case-by-case basis by interdisciplinary Heart Teams [3].

Given the limited number of patients available for study and the inherent heterogeneity within this patient cohort, it is not feasible to construct a systematic representation of the typical characteristics of individuals undergoing TAVI after OHT.

Furthermore, due to the presence of incomplete data within the identified literature and the restricted size of the population possessing this unique clinical profile, we were unable to perform a robust statistical analysis of the cases reported in the literature. Consequently, we adopted a descriptive approach, summarizing each case published up to the submission of our initial manuscript draft and presenting this information in a tabular format to enhance ease of comprehension.

The inaugural case of TAVI in an OHT recipient was published in 2010 by Seiffert and colleagues. This case involved an 81-year-old patient, deemed high-risk, who had undergone OHT 15 years prior. The patient exhibited a reduced left ventricular ejection fraction (LVEF) of less than 30%, attributed to concomitant coronary artery disease (CAD). The choice of the transapical approach was made due to severe calcification of the entire aorta and kinking of the iliac arteries. A 26 mm Edward Sapien transcatheter heart valve (THV) was utilized and successfully implanted, with minimal residual paravalvular regurgitation. Subsequently, the patient required a percutaneous coronary intervention (PCI) involving the placement of a drug-eluting stent (DES) in the left anterior descending (LAD) artery. Intraoperative transesophageal echocardiography (TEE) demonstrated improved contractility and an LVEF of 50%. Preoperative TTE had revealed a mean aortic gradient of 23 mm Hg and an effective orifice area of 0.6 cm^2^, consistent with the characteristic findings of low-flow, low-gradient severe aortic stenosis [6].

The second documented case, reported in the same year by Bruschi et al., involved a 67-year-old male patient who underwent TAVI for severe aortic stenosis approximately nine years after receiving a heart transplant. Preoperative assessments revealed significant findings, including a peak gradient of 87 mmHg, an indexed aortic valve area (AVA) of 0.5 cm^2^/m^2^, and pronounced left ventricular (LV) systolic dysfunction, with a LVEF of 35%. The medical team opted for a 29 mm CoreValve prosthesis (Medtronic) for the TAVI procedure, which resulted in only mild paravalvular regurgitation [7].

TAVI has also been employed as a treatment option for heart transplant recipients presenting with aortic regurgitation and deemed at high surgical risk. The initial case report of such an intervention was published by Zanuttini et al., involving a 75-year-old individual who had OHT approximately 14 years after the original transplantation procedure. The patient’s initial heart transplant had been necessitated by end-stage dilated cardiomyopathy (DCM) and had involved mitral and aortic valve replacements.

Preprocedural TTE revealed a mildly dilated left ventricle with moderate LVEF of 40% and thickening of the tricuspid aortic valve. The patient presented with severe aortic regurgitation characterized by a central jet, attributed to the deformation and retraction of the left coronary cusp. Given the patient’s high surgical risk, as indicated by a EuroSCORE of 36%, the medical team elected to perform a TAVI procedure. A 29 mm CoreValve prosthesis was employed for this intervention. Post procedure, the patient developed a third-degree atrioventricular (AV) block, necessitating the placement of a permanent pacemaker. Subsequent TEE demonstrated normal prosthetic valve function, with peak and mean gradients measuring 16 and 10 mmHg, respectively, and a mild paravalvular leak [8].

In 2013, Praetere et al. reported the fourth case involving a 77-year-old male patient who underwent TAVI for the treatment of aortic stenosis. This intervention took place two decades after the patient had received an OHT. Initial assessment of the patient revealed an aortic valve area (AVA) of 1 cm^2^, indicative of severe aortic stenosis. The patient also presented with a low-flow, low-gradient condition and severe left ventricular dysfunction, characterized by a left ventricular ejection fraction (LVEF) of less than 25%. Additionally, the patient had undergone multiple PCIs for CAD. Further diagnostic evaluation included low-dose dobutamine stress echocardiography (DSE), which was inconclusive in accurately assessing the true severity of aortic stenosis (AS). Consequently, a decision was made to perform a balloon aortic valvuloplasty (BAV) to reevaluate the patient’s symptoms and LVEF. In the months following the BAV procedure, the patient experienced moderate symptom improvement and a subsequent increase in LVEF to 30%. However, after seven months, the patient experienced symptom relapse, prompting the medical team to consider TAVI as the next course of action. The TAVI procedure was carried out via the transapical approach, utilizing a 23 mm Edwards Sapien valve. Postprocedural evaluation demonstrated a 30% LVEF and the absence of paravalvular leak, with peak and mean gradients measuring 18 and 11 mmHg, respectively [9].

The fifth case, documented by Ahmad et al. In 2016, involves a 25-year-old female who underwent an urgent OHT at the age of 11 due to severe congestive heart failure. The patient had previously undergone corrective surgeries for ventricular septal defects at ages 1 and 3. It’s worth noting that some cases of OHT lack comprehensive data regarding the dono’s heart at the time of implantation. However, in this particular case, it was reported that the donor was a 62-year-old woman with preexisting moderate coronary artery disease and mild aortic stenosis. Approximately 14 years following the initial heart transplant, the recipient began experiencing NYHA III heart failure symptoms. TTE revealed a severely calcified tricuspid aortic valve with severe aortic stenosis, characterized by a peak gradient of 77 mmHg and a mean gradient of 44 mmHg. The AVA measured less than 1 cm^2^, and severe aortic regurgitation was also present, despite normal LVEF. Subsequently, the patient underwent a TAVI procedure using a 23 mm Edwards Sapien 3 valve in a standard protocol. Following the TAVI intervention, the patien’s clinical condition improved significantly. Postprocedural TTE demonstrated normal valve function with no evidence of paravalvular regurgitation. Additionally, peak and mean gradients were measured at 20 mmHg and 11 mmHg, respectively, indicating favorable hemodynamic outcomes [10].

In the same year, Herrmann et al. reported another case of TAVI in an OHT recipient. This case involved a 73-year-old male who had received a heart transplant 13 years earlier and subsequently developed severe aortic stenosis, with the additional finding of a bicuspid aortic valve. Preprocedural TTE revealed an AVA measuring 0.58 cm^2^ and a mean gradient of 43 mmHg. Given the patient’s high surgical risk, as indicated by a Society of Thoracic Surgeons (STS) score of 8.024%, the decision was made to proceed with the TAVI procedure. The intervention followed the standard approach, utilizing a 26 mm Edwards Sapien 3 valve and the transfemoral access route. Postprocedural TTE assessment demonstrated the normal functionality of the implanted valve. Furthermore, there was no evidence of paravalvular regurgitation, and the measured peak and mean gradients were 27 mmHg and 13 mmHg, respectively, reflecting successful hemodynamic outcomes [11].

In 2018, Akleh et al. reported a case involving TAVI in an OHT recipient. The patient was a 77-year-old male who had undergone heart transplantation 23 years earlier and presented with exertional dyspnea attributed to aortic stenosis. The original heart transplant had been conducted due to idiopathic dilated cardiomyopathy (DCM), although no records were available from the 1994 procedure. The patient’s postoperative clinical course had been generally favorable, with the exception of paroxysmal atrial flutter, which necessitated the implantation of a single-chamber pacemaker in 2008. Subsequently, in 2010, the patient underwent atrial flutter ablation. TTE revealed progressive degeneration of a bicuspid aortic valve, indicated by a peak gradient of 65 mmHg, an AVA measuring 0.9 cm^2^, and a preserved LVEF of 59%. Multidetector computer tomography (MDCT) confirmed significant calcification of the aortic valve and ruled out aortoiliac atherosclerosis, which was crucial in selecting the transfemoral approach for the TAVI procedure. Given the patient’s high surgical risk, as denoted by a Society of Thoracic Surgeons (STS) score predicting a 7.035% mortality risk within 30 days, the Heart Team opted for TAVI as the preferred treatment strategy. The TAVI procedure adhered to established protocols and utilized a 29 mm Edwards Sapien 3 valve. Additionally, the patient’s single-chamber pacemaker was upgraded to a dual-chamber system on the day following the TAVI. Postprocedural TTE revealed normal function of the aortic bioprosthesis, with peak and mean gradients measuring 14 mm Hg and 12 mm Hg, respectively. No paravalvular leak was observed, and the patient maintained a preserved LVEF. Following the procedure, the patient experienced symptomatic and hemodynamic improvement and was discharged 48 h post TAVI. He continued to receive immunosuppressive therapy as part of his ongoing post-transplant care [12].

In 2019, Avula S. et al. reported the eighth case of TAVI in an OHT recipient in the United States. This case involved a 73-year-old male who had undergone heart transplantation 19 years prior due to non-ischemic cardiomyopathy. Over the years following the transplant, a transthoracic echocardiogram revealed progressive sclerotic changes that began around 15 years post-transplantation and eventually led to the diagnosis of severe aortic stenosis in 2019. The patient’s post-transplant medical history indicated the presence of non-occlusive CAD in addition to aortic stenosis. Given the patient’s high surgical risk, as indicated by an STS score of 12.20%, the medical team opted for TAVI as the preferred treatment strategy. The TAVI procedure was conducted in a standard manner, utilizing a 29 mm Edwards Sapien 3 valve. Local anesthesia and sedation were employed for the procedure. Postprocedural TTE demonstrated the normal functioning of the implanted valve, with no evidence of valve leakage, and the patient exhibited a normal LVEF. Following the intervention, the patient’s clinical course was favorable, and he was discharged on the second day after the procedure [13].

In 2020, Beale et al. reported the ninth case of TAVI in OHT recipients. This particular case involved a 45-year-old patient who had received a heart transplant 22 years earlier, originally due to non-ischemic cardiomyopathy. The patient’s medical history included several notable factors, such as a BAV in the transplanted heart, hypertension, hyperlipidemia, and various transplantation-associated complications. Among the notable complications were squamous cell carcinoma of the hard palate, which had been treated with resection and grafting, as well as end-stage renal disease (ESRD) necessitating hemodialysis due to cyclosporine toxicity. Given the patient’s complex medical history and high-risk status, the Heart Team elected to perform a TAVI procedure, which yielded optimal results [14].

The tenth case reported involved a 61-year-old patient who developed aortic stenosis a remarkable 34 years after undergoing an OHT. Unfortunately, there were no available details regarding the specific type of heart donor used in the transplantation. The patient’s advanced aortic stenosis was treated with a self-expandable valve due to the presence of a significantly enlarged annulus and severe calcifications affecting both the mitral and aortic valves. The TAVI procedure yielded favorable outcomes, as evidenced by the absence of valve leakage and the preservation of a normal LVEF [15].

In Table 1, we have systematically summarized the findings to facilitate a comparative analysis of common characteristics between the case reports and the evidence found in the literature.

## 6. Case Presentation

We present the case of a 62-year-old male with a history of OHT a decade ago. He had a medical history that included a myocardial infarction with delayed admission, leading to refractory cardiogenic shock necessitating an intra-aortic balloon pump (IABP) and veno-arterial extracorporeal life support (V-A ECLS). Due to his critical condition, he underwent emergent OHT, resulting in a NYHA class II heart failure diagnosis and moderate aortic stenosis. Limited information was available about the donor, except that the allograft came from a marginal donor.

Upon discharge, the recipient had an aortic valve area of 1.1 cm^2^, but no data were available regarding transvalvular gradients. The patient also had chronic kidney disease (creatinine 1.75 mg/dL), colonic diverticulosis, hyperuricemia, and a right bundle branch block. Several months before admission, the patient experienced flu-like symptoms, which were not confirmed as a COVID infection.

His current home treatment regimen included antiplatelet therapy, a low-dose diuretic, cholesterol-lowering medication, and immunosuppressive therapy (cyclosporine, prednisone, and mycophenolic acid). Unfortunately, the patient did not adhere to the mandatory follow-up plan for cardiac transplant recipients, and there had been no recent bloodwork or cardiac evaluations in the past five years.

Upon admission, TTE revealed a LVEF of 40% with mild diffuse hypokinesia. Additionally, there was evidence of degenerative aortic disease with severe stenosis and moderate aortic regurgitation, accompanied by substantial calcifications of the aortic leaflets. The AVA measured 0.4 cm^2^, with a peak gradient of 100 mmHg, a mean gradient of 62 mmHg, and a 20 mm diameter aortic annulus. The patient exhibited moderate concentric hypertrophy, severe diastolic dysfunction with a restrictive pattern, and elevated filling pressures. Left atrium dilation was notable, with a volume of 140 mL. The evaluation of other valves indicated mild degenerative mitral regurgitation with posterior annulus calcifications, as well as moderate tricuspid and pulmonary regurgitations. The right ventricle displayed normal contractility, but the estimated pulmonary artery systolic pressure (PASP) was 69 mmHg. A further CT-angiography (CTA) scan indicated a dilated ascending aorta with a diameter of 41 mm.

The Heart Team reviewed the case, taking into account the patient’s immunosuppressed status and the high risk associated with a redo intervention. The decision was made to proceed with TAVI. The patient underwent the necessary pre-procedural assessments, including coronary angiography, CTA scans, and bacteriology and viral screenings.

**Figure 1:**
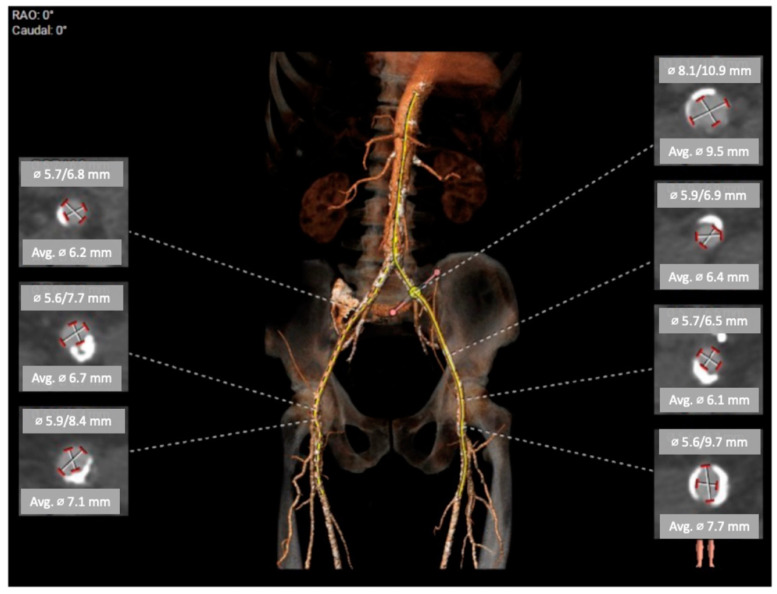
CTA with 3D reconstruction—access vessels.

**Figure 2:**
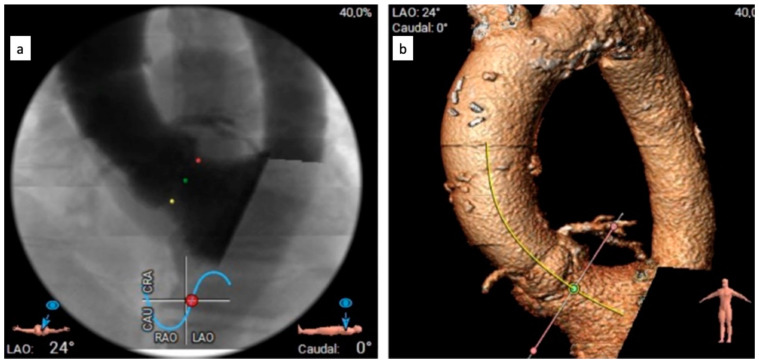
Investigative images: Aortography—aortic root and ascending aorta, and origins of coronary arteries (**a**) and CTA 3D reconstruction—aortic root with coronary arteries origin, ascending aorta (**b**).

The CTA scan revealed a tricuspid aortic valve with an annulus area of 434 mm^2^ and dimensions near the lower limit, measuring between 26 mm and 23 mm. Notably, calcium protrusions toward the left ventricular outflow tract (LVOT) were observed below the left coronary cusp (LCC) and non-coronary cusp (NCC), increasing the risk of disturbances. To determine sizing and assess calcium behavior, a balloon valvuloplasty was considered. The left coronary artery (LCA) was found to be at a distance of 15 mm from the aortic annulus, while the right coronary artery (RCA) was at a distance of 17.5 mm. The access vessels showed calcification, with borderline diameters for eSheath placement on both sides. CTA with 3D reconstruction—access vessels is presented in Figure 1. In Figure 2 can be observed aortography of aortic root and ascending aorta, and origins of coronary arteries (a) and CTA 3D reconstruction of aortic root with coronary arteries origin, ascending aorta (b). The lines in Figure 2a represent the plane of the aortic annulus (pink) and the position perpendicular to the aortic axis (yellow). Proper alignment for prosthesis implantation

The TAVI procedure was performed under general anesthesia and standard monitoring protocols, with temporary rapid pacing achieved through an internal jugular vein catheter.

**Figure 3:**
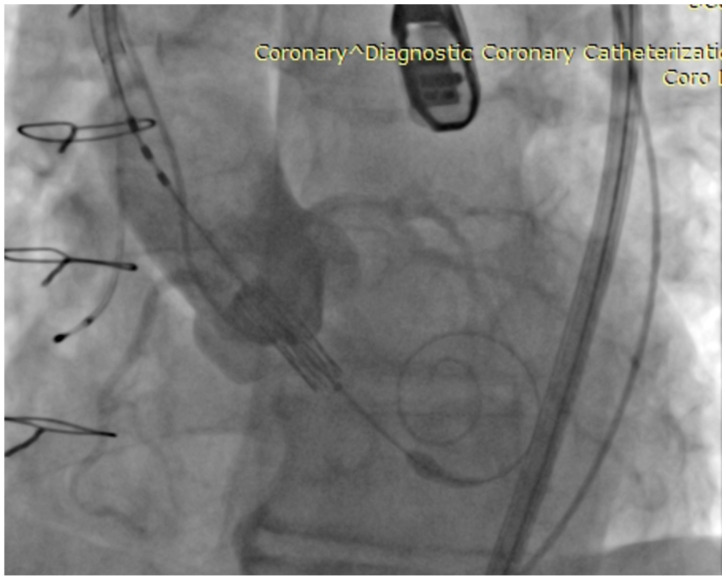
Fluoroscopy image—Valve positioning.

Percutaneous diagnosis was accomplished using a 6 French (F) sheath inserted via the left femoral artery. Subsequently, a Safari stiff guidewire was introduced, followed by the insertion of a specific eSheath through the right femoral artery. The hydrophilic guidewire, along with the AL 1 catheter, was navigated retrogradely across the aortic valve. Following this, the hydrophilic guidewire was exchanged for the stiff Safari guidewire, and the Sapien 3 valve, size 23 mm, was positioned at the level of the aortic annulus. Figure 3 presents a fluoroscopy image showing valve positioning. With the aid of rapid pacing, the valve was expanded (nominal volume +1 mL). During transesophageal echocardiography (TEE) assessment, a paravalvular leak was detected.

**Figure 4:**
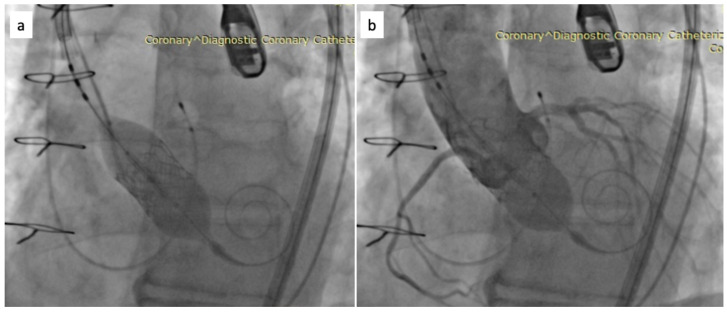
Fluoroscopy images of aortic root—baloon inflating in order to expand the aortic prosthesis (**a**) and inflated baloon in the expanded prosthesis with coronary arteries visible (**b**).

**Figure 5:**
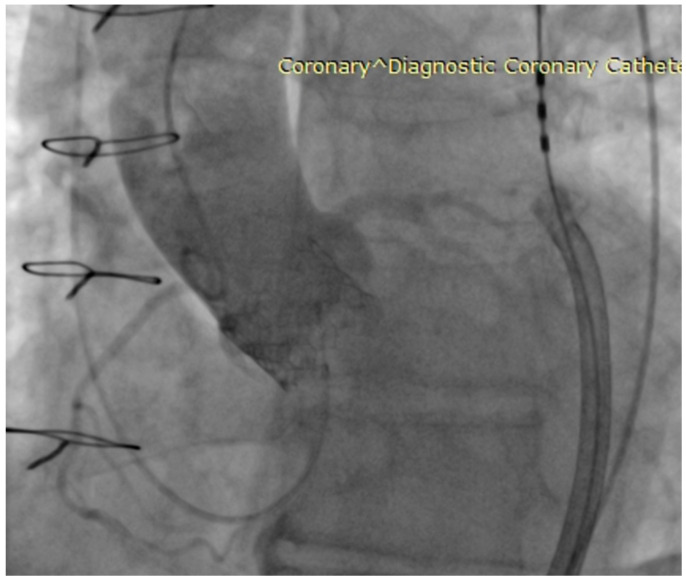
Fluoroscopy image—Checking the valve position and regurgitation.

To address the paravalvular leak, a subsequent post-dilation was performed through inflating the valve’s specific balloon with a nominal volume of +2 mL. Fluoroscopy image of aortic root showing baloon inflating in order to expand the aortic prosthesis is observed in Figure 4a, while Figure 4b presents a fluoroscopy image showing inflated baloon in the expanded prosthesis with coronary arteries visible. TEE examination following the post-dilation revealed minimal regurgitation. At the supravalvular aortic injection, only mild regurgitation was observed. Figure 5 presents a fluoroscopy image where checking the valve position and regurgitation can be observed. The guidewires were then carefully withdrawn, and another TEE confirmed minimal residual regurgitation. Hemostasis was achieved through the use of Proglide and Angioseal devices following the removal of guidewires and sheaths.

The patient experienced an uneventful recovery, marked by extubation within one hour of the procedure. On the second day, the patient was transferred to the ward, and after one week, he was discharged. Upon discharge, the patient displayed no symptoms of heart failure and exhibited improved cardiac function. During the first follow-up appointment at one month post procedure, mild aortic regurgitation was detected, though it was not associated with significant symptoms.

At the time of discharge, the patient received the following medical treatment recommendations per day:

- 100 mg aspirin;
- 150 mg cyclosporine;
- 1440 mg mycophenolate;
- 5 mg prednisone;
- 150 mg allopurinol;
- 40 mg famotidine;
- 40 mg furosemide;
- 10 mg atorvastatin;
- 160 mg fenofibrate.

These medications were prescribed to optimize the patient’s post-procedure care and management.

## 7. Discussion

The enhanced categorization of heart failure is imperative for optimizing the timing of surgical interventions, particularly in critical and emergent scenarios. A significant development in this regard occurred in 2009 with the publication of a study that introduced the INTERMACS classification (Interagency Registry for Mechanically Assisted Circulatory Support). This classification system delineates seven distinct clinical profiles of heart failure, offering a valuable framework for refining treatment strategies in advanced heart failure cases.

Of particular relevance are Classes 1 and 2 within the INTERMACS classification, as patients falling into these categories require urgent interventions such as assist device implantation or OHT. In these critical cases, there arises an opportunity to expand the pool of available donor organs through considering a supplementary list of marginal donors. This approach aims to provide patients with a lifeline, thereby reducing the risk of mortality while awaiting transplantation [16]. Classes 1 to 3 encompass patients who are currently reliant on inotropic agents and are in pressing need of heart transplantation. It is noteworthy that a substantial majority, approximately 80%, of heart transplantations are conducted within this subset of heart failure patients. Furthermore, there is a burgeoning consensus within the medical community, particularly for patients falling under the INTERMACS Classes 1 and 2, in favor of considering hearts from marginal donors for transplantation. This shift in perspective acknowledges the critical urgency of addressing the needs of these high-risk patients and underscores the potential benefits of utilizing marginal donor organs to save lives in these challenging cases [17].

Given the rising demand for heart transplantation and the elevated mortality risk associated with patients classified under Class 1 and Class 2 heart failure, numerous transplant centers are confronted with the necessity of considering hearts that may not meet the traditional criteria for ideal transplantation candidates. This includes the potential acceptance of hearts from older donors with conditions such as moderate valvular disease, mild coronary artery disease, and comorbidities like hypertension, diabetes, and dyslipidemia. These deviations from the strict transplantation contraindications reflect the imperative to expand donor options in order to address the pressing need for life-saving heart transplants [3].

In select emergency cases, transplant centers may find it necessary to accept hearts from marginal donors, even if the results may be less than ideal. This decision may entail making certain annotations to the donor criteria. Notable considerations include:

- Increase in donor age: The acceptance of older donors, acknowledging that chronological age alone may not necessarily preclude organ suitability.
- Extended ischemic time: A willingness to tolerate longer periods of ischemia, recognizing that timely organ retrieval may not always be feasible.
- Acceptance of size mismatch: Being open to heart size variations between donor and recipient, as long as the functional compatibility is maintained.
- Atrioventricular valvular disease: The inclusion of donors with atrioventricular valvular disease, provided it does not compromise overall heart function.
- Left ventricular hypertrophy: The consideration of hearts with left ventricular hypertrophy, which may be compatible with transplantation in certain cases.
- Hepatitis B/C: The acceptance of hearts from donors with hepatitis B or C, while carefully evaluating the recipient’s condition and risk factors.
- Significant coronary artery disease: The evaluation of donor hearts with significant coronary artery disease, taking into account the potential impact on transplantation success.
- Brain malignancies: The assessment of hearts from donors with brain malignancies, weighing the risks and benefits for recipients in critical need.
- Non-beating hearts: The consideration of non-beating hearts (donors who have experienced cardiac arrest), contingent upon the viability of the organ.
- Drug abuse/intoxication: The evaluation of hearts from donors with a history of drug abuse or intoxication, with careful consideration of the recipient’s circumstances.

Each of these characteristics presents potential independent risk factors for the outcome of OHT. However, in cases involving terminally ill patients with no alternative treatment options, accepting hearts from donors with these characteristics may represent a viable solution, potentially leading to an extended lifespan and improved quality of life (QOL) for the recipient [3].

The concept of implementing alternative donor lists for marginal donors has arisen as a means to expand the pool of potential donor options. It is estimated that through increasing the age limit for donors to 55 years old and potentially even up to 65 years old, more than 15,000 patients could potentially benefit from OHT. In the case of the higher age limit, the number of potential beneficiaries could range from 50,000 to 70,000, assuming that the survival rates align with current research findings. The acceptance of alternative lists for marginal donors hinges on the condition that the survival rates achieved with these donors remain comparable to the existing standards. Similar to the strategies employed in kidney transplantation, where “old-to-old” donor–recipient pairings have been successful, embracing hearts from marginal donors could provide the opportunity to include older, critically ill patients on the transplant waiting list. This approach seeks to maximize the utilization of available donor organs while prioritizing the well-being and survival of transplant recipients [3].

The International Thoracic Organ Transplant Registry of the International Society for Heart and Lung Transplantation (ISHLT) has compiled data from a total of 481 adult thoracic transplant centers across the globe. This dataset is estimated to encompass approximately 75% of all heart transplants performed worldwide. The ISHLT has leveraged this comprehensive data to analyze changes in recipient characteristics over recent decades. According to the ISHLT’s 2021 data publication, the landscape of heart transplant recipients has evolved significantly. This transformation can be attributed to advancements in mechanical circulatory support therapies, improved medical and surgical treatments, and modifications to the criteria for accepting candidates for heart transplantation. Consequently, older and sicker patients have been included in the transplant population and have benefited from appropriate medical care. The study conducted by the ISHLT spans a substantial timeframe, covering the years from 1992 to 2018, and includes data from three major regions: North America, Europe, and other countries, encompassing South America, Asia, the Middle East, Australia, and various other regions. This comprehensive analysis sheds light on the dynamic changes in heart transplant recipient demographics and highlights the evolving landscape of heart transplantation on a global scale [18,19,20].

To analyze the trend in recipient acceptance criteria, the study divided the data into three distinct periods: 1992–2000, 2001–2009, and 2010–2018, with a similar number of heart transplants performed in each period. Over this time frame, several noteworthy changes in recipient characteristics were observed:

- Increasing age: Recipient age has seen a steady rise, increasing from an average of 53 years in the first period to 57 years in the most recent period.
- Higher body mass index (BMI): The median BMI has also increased, with values rising from 25 kg/m^2^ to 26.5 kg/m^2^. This shift is attributed to a corresponding increase in median weight, from 75.7 kg to 80 kg.
- Kidney function trends: Surprisingly, there has been a declining trend in kidney dysfunction, as measured via glomerular filtration rate (GFR), across all three geographic regions. Several factors contribute to this trend, including an increased incidence of heart–kidney transplants for candidates with severe kidney disease, improved kidney function resulting from the use of ventricular assist devices before transplant, a decreasing incidence of recipients with ischemic cardiomyopathy (who are more prone to kidney disease due to cardiovascular risk factors), and a rising percentage of female recipients.
- Blood type compatibility: The blood type of recipients has remained relatively stable over time. This is attributed to the increase in transplantation of allo-sensitized patients, likely due to advances in managing patients before and after transplantation. These advances include therapies for pretransplant desensitization and posttransplant rejection, improved human leukocyte antigen (HLA) typing, enhanced antibody detection and quantification. Consequently, the percentage of patients with a pretransplant panel of reactive antibodies (PRA) exceeding 20% increased from 5.2% to 17.9%, and the percentage of highly sensitized patients (PRA > 80%) rose from 0.9% to 3.6% over the same period.
- Increased medical comorbidities: Heart transplant candidates have shown a greater burden of medical comorbidities over time. This includes a rising incidence of diabetes (from 16.7% to 27%), a history of malignancy (from 3.8% to 8.7%), previous cardiac surgery (from 37.8% to 50.1%), and dialysis (from 3% to 4.7%). The increase in dialysis cases is often associated with non-acute kidney failure and a higher likelihood of recovery. Additionally, patients with chronic kidney disease on dialysis may be referred for simultaneous heart–kidney transplantation.

These trends highlight the evolving landscape of heart transplant recipient characteristics, reflecting changes in both the patient population and the criteria for heart transplant candidate acceptance over the decades [19].

During the same period from 1992 to 2018, Khush et al. conducted an analysis of donor data and observed several trends in how the donor profile has evolved. These trends include:

- Increased donor age: The median age of donors has seen a notable increase worldwide, rising from 31 years to 35 years. This trend is partly attributable to changes in transplant practices in Europe, where the median donor age has significantly increased from 31 to 45 years.
- Higher body mass index (BMI): Donor BMI has also experienced an increase, with the median BMI rising from 24.1 kg/m^2^ to 26 kg/m^2^. This change is likely associated with the growing prevalence of obesity, primarily observed in North America.
- Stable gender distribution: The distribution of donor genders has remained relatively stable over time, with approximately 70% of donors being male and 30% female.

These trends in donor characteristics provide insights into the shifting donor profile over the years, reflecting changes in both donor demographics and health-related factors [20].

An intriguing observation is that the cause of death among donors in the European region has shifted from predominantly head trauma to a higher incidence of cerebrovascular accidents (CVAs) or strokes. Conversely, in North America, head trauma remains the primary cause of donor death, but there is also an increase in deaths due to anoxia, which could be linked to a rise in opioid use and drug abuse.

The increasing age of donors in Europe may play a role in this change in the cause of death, as older donors are more likely to experience natural and age-related deaths. This shift in donor causes of death highlights the complex interplay of various factors, including medical conditions, lifestyle choices, and demographics, that can influence the donor pool and impact the availability and suitability of donor organs for transplantation [20].

Observations regarding substance use among donors have revealed notable trends over the years:

- Cigarette smoking: The percentage of donors with a history of cigarette smoking (≥20 pack-years) has decreased significantly, dropping from 40% in 1995 to 15% in 2018. This decline likely reflects broader efforts to reduce smoking rates and improve public health.
- Alcohol use: Donor alcohol use (≥2 alcoholic drinks per day) has remained relatively stable during this period, suggesting that alcohol consumption patterns among donors have not undergone significant changes.
- Cocaine use: There has been a troubling increase in cocaine use among donors, rising from 11% in 2000 to 27% in 2018. This increase in cocaine use raises concerns about the potential health implications for both donors and recipients.
- Use of other drugs: Donors reporting the use of other drugs, such as non-intravenous street drugs like crack, marijuana, prescription narcotics, sedatives, hypnotics, or stimulants, have seen a substantial increase.

This percentage surged from 25% to 57% over the same period. This significant rise in the use of other drugs underscores the need for careful donor screening and evaluation to ensure the safety and health of both donors and transplant recipients.

These trends in substance use among donors highlight the evolving landscape of donor health behaviors and underscore the importance of robust donor screening protocols to safeguard the well-being of transplant recipients [20].

Despite advancements in organ procurement and preservation, increased willingness to accept marginal donor hearts, and the utilization of hearts from donors with certain medical conditions (such as drug abuse or hepatitis C viremia) and after circulatory death, the number of heart transplants has remained stable over this period. This stability in the number of heart transplants suggests that there may be limitations to increasing the rate of procedures, primarily due to a shortage of available donor organs [19,20].

The availability of suitable donor organs remains a critical factor in determining the number of heart transplants that can be performed. Efforts to address this limitation may include initiatives to increase organ donation rates, improve donor organ quality, and enhance organ preservation techniques. The stability in the number of heart transplants underscores the ongoing challenges in meeting the demand for life-saving heart transplant procedures [19,20,21,22].

## 8. Conclusions

Despite advancements in organ procurement and preservation, increased willingness to accept marginal donor hearts, and the utilization of hearts from donors with certain medical conditions (such as drug abuse or hepatitis C viremia) and after circulatory death, the number of heart transplants has remained stable over this period. This stability in the number of heart transplants suggests that there may be limitations to increasing the rate of procedures, primarily due to a shortage of available donor organs.

The availability of suitable donor organs remains a critical factor in determining the number of heart transplants that can be performed. Efforts to address this limitation may include initiatives to increase organ donation rates, improve donor organ quality, and enhance organ preservation techniques. The stability in the number of heart transplants underscores the ongoing challenges in meeting the demand for life-saving heart transplant procedures.

In conclusion, the growing need for improved treatment options in the medical field has driven physicians to explore innovative methods that were once considered unthinkable. TAVI stands as a notable example of medical progress throughout history. Today, there is a rapid increase in the demand for effective treatments for end-stage heart failure, which has necessitated a reevaluation of the criteria for “ideal” donors.

Despite continuous advancements in assist devices, heart transplantation remains the ultimate solution for many patients. Expanding the donor pool has become imperative in addressing this increasing need. However, the inclusion of marginal donors presents new challenges in treating organs that may have previously been compromised.

TAVI has emerged as a proven and versatile treatment method, particularly for high-risk patients like heart transplant recipients. It has become one of the primary tools in managing the complexities associated with the use of marginal donors. Our case presentation contributes to addressing the current gap in documentation regarding the use of TAVI in heart transplant recipients. Coupled with a brief literature review, it underscores the feasibility and reliability of TAVI as a valuable solution within this specific medical context.

## Figures and Tables

**Table 1:** Literature review summary of the TAVI procedure in OHT patients.

Authors	Year Published	Age at Transplant	Age at TAVI (Years)	Years from OHT to TAVI	LVEF (%)	TVG (mmHg)	TAVI Valve Size (mm)	Type of TAVI	Type of Implantation	Outcome
Seiffert et al. [6]	2010	66	81	15	<30	M23	26	Edward Sapien	TA	DES/LAD; LVEF 50%
Bruschi et al. [7]	2010	56	67	9	35	P87	29	CoreValve	TF	Mild paravalvular regurgitation
Zanuttini et al. [8]	2013	61	75	14	40	No data	29	CoreValve	TF	Mild paravalvular regurgitation 3rd-degree AV block, Permanent pacemaker
Praetere et al. [9]	2013	57	77	20	<25%	32/17	23	Edward Sapien	TA	LVEF 30%; no paravalvular leak
Ahmad et al. [10]	2016	11	25	14	Normal	77/44	23	Edward Sapien 3	TF	no paravalvular leak
Julien et al. [11]	2016	60	73	13	No data	M43	26	Edward Sapien 3	TF	no paravalvular leak
Akleh et al. [12]	2018	54	77	23	59	P65	29	Edward Sapien 3	TF	No paravalvular leak
Avula S. et al. [13]	2019	54	73	19	Normal		29	Edward Sapien 3	TF	No paravalvular leak
Beale et al. [14]	2020	23	45	22	Normal	M52	No data	No data	TF	complete heart block, trace paravalvular leak
Saad M. Ezad et al. [15]	2022	27	61	34	No data	No data	34	Evolut R	TF	No paravalvular leak
